# Radiotracer labeled thymohydroquinyl gallate capped gold nanoparticles as a theranostic radiopharmaceutical for targeted antineoplastic and bioimaging

**DOI:** 10.1016/j.jpha.2024.100965

**Published:** 2024-03-12

**Authors:** Munaza Batool, Batool Fatima, Dilshad Hussain, Rubaida Mahmood, Muhammad Imran, Saeed Akhter, Muhammad Saqib Khan, Saadat Majeed, Muhammad Najam-ul-Haq

**Affiliations:** a Department of Biochemistry, Bahauddin Zakariya University, Multan, 60800, Pakistan; b HEJ Research Institute of Chemistry, International Center for Chemical and Biological Sciences, University of Karachi, Karachi, 75270, Pakistan; c MINAR Cancer Hospital Multan, Multan, 60000, Pakistan; d Biochemistry Section, Institute of Chemical Sciences, University of Peshawar, Peshawar, 25120, Pakistan; e Institute of Chemical Sciences, Bahauddin Zakariya University, Multan, 60800, Pakistan

**Keywords:** Cancer theranostics, Gallic acid, Gold nanoparticles, Targeted drug delivery, Thymoquinone

## Abstract

Thymoquinone (TQ) and gallic acid (GA) are known for counter-tumorigenic characteristics. GA inhibits cancer cell proliferation by interfering with many apoptotic signaling pathways, producing more reactive oxygen species (ROS), focusing on the cell cycle, and suppressing the expression of oncogenes and matrix metalloproteinases (MMPs). In this study, TQ (after reducing to thymohydroquinone) and GA are esterified to form thymohydroquinyl gallate (a prodrug). Thymohydroquinyl gallate (THQG) possesses enhanced antineoplastic efficacy and targeted delivery potential. The chemical and spectroscopic analysis confirms ester synthesis. Gold nanoparticles (AuNPs) are employed as nanocarriers due to their physicochemical and optical characteristics, biocompatibility, and low toxicity. As an efficient drug transporter, (AuNPs shield conjugated drugs from enzymatic digestion. The prodrug acts as a reducing agent for Au metal atoms and is loaded onto it after reduction. The nano drug is radiolabeled with ^99m^Tc and ^131^I to monitor the drug biodistribution in animals using a gamma camera and single-photon emission computerized tomography (SPECT). ^131^I is an antineoplastic that helps enhance the drug's efficiency. Chromatographic results reveal promising radiolabeling percentages. *In vitro,* drug release shows sustained release at pH ⁓5.8. *In vitro* 3-[4,5-dimethylthiazol-2-yl]-2,5 diphenyl tetrazolium bromide (MTT) cytotoxicity assay reveals drug potency on CAL 27 and MCF 7 cell lines.

## Highlights

• Evaluation of dual antineoplastic role by combining Thymoquinone and Gallic Acid.
• Bioimaging of drug biodistribution by radiolabeled prodrug composite.
• Targeted delivery of prodrug conjugate to cancer cells *in vitro* and in vivo.
• Comparative studies are carried out to assess the impact of Thymoquinone modification.

## Graphical abstract

**Figure:**
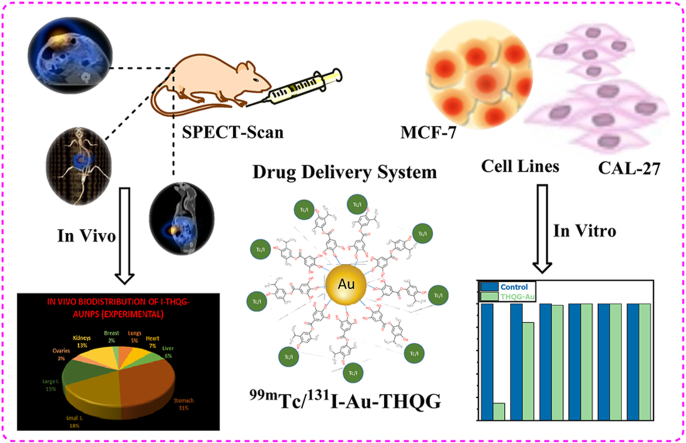

## 1 Introduction

Uncontrolled cell proliferation leads to cancer; these aberrant cells can spread to or invade other body regions. Cancers are divided into lymphoma, sarcoma, leukemia, carcinoma, germ cell tumor, and blastoma based on the alleged origin of tumor cells. Developing drug delivery methods allows for adjusting the drug's pharmacological properties without sacrificing the desired impact on molecular targets. Notably, they can alter a drug's pharmacokinetics, absorption, stability, and exposure to tumors and healthy tissues, making drug administration easier.

*Nigella sativa* (*N. sativa*) is a yearly indigenous herb found in South Asia, North Africa, and Europe. *N.* *sativa* extract contains thymoquinone (TQ) as the primary pharmaceutical compound, showing hepato-protective, antioxidant, and anti-inflammatory effects [1,2]. Natural compounds like Tq have been explored for anti-carcinogenic bioactivity in multiplex neoplasms with restricted cytotoxicity towards healthy tissues. TQ inhibits the tumor cells' growth by disrupting microtubule organization, triggering a cell cycle halt and downregulation of cellular protein expression. In colon cancer, human breast cancer, and osteosarcoma cells, TQ can arrest the G1 phase of the cell cycle by suppressing the activation of cyclin E or cyclin D and upregulating p21 and p27 (a cyclin-dependent kinase (Cdk) inhibitor). Targeting a specific cell cycle phase largely depends on TQ concentration. In human breast cancer (MCF-7 cell lines), a high TQ level promotes the G2 phase arrest, and a low TQ concentration arrests the S phase [[3], [4], [5]].

Gallic acid (GA) is one of the most significant plant polyphenols with health-promoting properties, treating viral and bacterial infections, cancer, inflammation, cognitive disorders, gastrointestinal disorders, and metabolic diseases. GA prevents bacterial growth by changing their membrane texture, impairing their metabolism, and preventing biofilm development. Additionally, GA inhibits the cancer cells' proliferation by interfering with many apoptotic signaling pathways, producing more reactive oxygen species (ROS), and suppressing the expression of matrix metalloproteinases (MMPs) and oncogenes [6].

Blood, urine, and feces also contain GA, possessing anti-inflammatory, antiallergic, anticarcinogenic, and antioxidant properties [[7], [8], [9]]. The antineoplastic role of GA in various neoplasia has been assessed with underlying genetically controlled mechanisms. Studies on cancers, including breast cancer cells, lymphoma, colon cancer, oral cancer, melanoma, and leukemia, have shown that GA and its derivatives exhibit anticancer properties. GA and its ester derivatives possess antineoplastic ability and selective cytotoxicity against neoplastic cells, demonstrating greater activity towards tumor cells than the normal ones [10]. An ester prodrug of TQ and GA is prepared after sodium borohydride reduction of TQ to thymohydroquinyl (THQ), involving the C=O bond conversion to C–OH [11]. The esterification produces thymohydroquinyl gallate (THQG) [12], demonstrating an enhanced anticancer effect via the dual antineoplastic role of TQ and GA. Lower pharmacologically optimum concentrations can be achieved via targeted drug delivery (TDD), significantly cutting the drug dosages. A TDD approach can also dramatically reduce side effects and damage to healthy cells and tissues. Therefore, creating novel TDD based on nanotechnology and combination therapy can potentially reduce the adverse effects of conventional antineoplastic approaches.

Bio-nanotechnology integrates biomolecules into nano-constructs to treat various human diseases [13]. Recent research has demonstrated the effectiveness of nanomaterials for neoplasm targeting, imaging, and therapeutic purposes. Nanoparticles (NPs) can also enshroud genes, medicines, and imaging molecules. Nano-drug delivery systems involve nanocarriers for natural or synthetic drug delivery through interior or exterior chemisorption [14,15]. The main obstacle in the practical application of nanoscale therapies is their low tumor-specific absorption. NPs can enter the normal cells and deliver high dosages directly into tumor cells. Despite the lack of clarity surrounding the mechanism(s) involved, NPs can readily penetrate cells. Over the past few years, much work has been done on nanotechnology-based products, including micelles, dendrimers, liposomes, polymers, and inorganic NPs. These NPs show superior performance to traditional therapies due to large surface-to-volume ratio and small size. Gold nanoparticles (AuNPs) can bio-image the neoplasms and be employed in drug delivery. They have a binding affinity for biochemical structures with large surface area and uniform dispersity for cancer theranostic with negligible toxicity [16]. Researchers are paying increased attention to AuNPs as drug delivery systems due to their lower toxicity, higher biocompatibility, hydrophilicity, and improved cell absorption. NPs-based TDD can efficiently deliver drugs to targeted tissues, enhance bioavailability and solubility, reduce toxicity, enhance medication efficacy, and shield therapeutic agents from deterioration. Synthetic and plant-derived polyphenols are capping agents for colloidal AuNPs [17]. Herein, the ester prodrug is used as a reducing or capping agent for AuNPs synthesis (resulting in drug loading). Finally, oval-shaped synthetic polyphenolic ester-capped colloidal AuNPs are prepared as excellent theranostic material called THQG-capped AuNPs (THQG−AuNPs). AuNPs exhibit high surface area, dense drug loading, and extensive cell penetration due to biocompatibility and nanosize, accounting for TDD of ester prodrug to cancerous cells. A well-known phenomenon for solid tumors is the enhanced permeability and retention (EPR) effect, wherein the deformed blood vessels and comparatively underdeveloped lymphatic systems cause a passive buildup of NPs and macromolecules. This prodrug composite exhibits an EPR effect, sustained drug release, and sustained therapeutic role in tumor microenvironments.

Radiolabeling can visualize the drug along the metabolic route in animals. Radioisotopes, ^99m^Tc and ^131^I are utilized to prepare radiolabeled complexes of THQG−AuNPs, i.e., ^99m^Tc−THQG−AuNPs and ^131^I−THQG−AuNPs for tracked visualization of drug biodistribution, taking advantage of ^99m^Tc and ^131^I both being gamma-emitting radionuclides. ^131^I (*t*_1/2_ = 8.01 days) undergoes gamma emission up to 364 keV and beta emission up to 0.608 keV. ^131^I is an antineoplastic that helps enhance the drug efficiency, similar to internal radioisotope therapy (RIT). Improved drug efficiency and RIT-induced EPR effect in TDD resulted from ^131^I−THQG−AuNPs where TQ and GA doubled the antineoplastic activity at the tumor site. Another pH sensitivity of novel nano-composite is also explored, maintaining sustainable release in tumor microenvironment (pH = 5.8). A combined multiplex novel technique is used to formulate a novel targeted and visualizable antineoplastic drug based on potent anticancer components of natural plant sources to address various existing problems and hurdles of conventional chemotherapeutics and radiotherapies in treating malignancies and tumors. This strategy comprising novel drug synthesis can be explored with other new combinations of individual drugs to enhance TDD.

## 2 Materials and methods

### 2.1 Reagents

Thymoquinone (TQ, ≥ 98%), gallic acid (GA, 97.5%), sodium hydrogen phosphate, (Na_2_HPO_4_, 99.95%), monosodium phosphate (NaH_2_PO_4_,≥ 98%), N-bromosuccinimide (NBS, ⁓99%) and sodium thiosulphate (Na_2_S_2_O_3_, ⁓98%) were bought from Sigma Aldrich (St. Louis, MI, USA). Sodium borohydride (NaBH_4_, 37.84 g/mol) and stannous chloride dihydrate (SnCl_2_·2H_2_O, ≥ 99%) were purchased from Daejung (Busan, Korea). Technetium (^99m^Tc,≥ 95%) and iodine (^131^I, 5mCi/10 μL) were obtained from MINAR Cancer Hospital (Multan, Pakistan). Gold(III) chloride (HAuCl_4_·3H_2_O, ⁓99%) was bought from MERCK (Rahway, NJ, USA).

### 2.2 Methods

#### 2.2.1 Synthesis of THQG

##### 2.2.1.1 Reduction of TQ into THQ using NaBH_4_

**Scheme 1:**
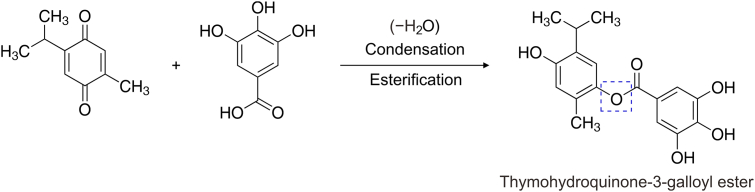
General reaction of prodrug thymohydroquinyl gallate (THQG) synthesis.

200 mg TQ and 1600 μL ethanol were added to a 25 mL flask. The mixture was swirled gently for several minutes at room temperature. 40 mg NaBH_4_ was added in small portions in 5 min and stirred for another 20 min. The mixture was cooled in an ice water bath, and 120 μL of 6 M HCl and 2 mL distilled water were added. Finally, 1 mL of distilled water was added after 15 min to quench the reaction. The product was collected by vacuum filtration, washed with ice-cold water, and dried for 15 min. The reaction scheme is given in Scheme 1.

NaBH_4_ provides hydride ions (H^−^) to reduce aldehydes and ketones. The core boron atom in NaBH_4_ is surrounded by four hydrogen atoms in a tetrahedral structure with a formal charge of −1. However, the negative charge on boron does not signify a lone pair on the element. The electrons in the B–H bond are polarised towards hydrogen since hydrogen has a higher electronegativity (2.20) than boron (2.04). Thus, the B–H bonds are polarised in the direction of hydrogen. Despite the formal negative charge, the hydrogen atoms carry partial negative charges and are nucleophilic. Quinone formation mainly involves two steps.

In the first step, nucleophilic attack of BH_4_^−^ anion occurs on both carbonyl carbons of TQ designated as carbonyl carbons a and b. Here, the double bond of carbonyl carbons is broken, BH_2_ is attached to the carbonyl center, and negative charges appear. Subsequently, an intermediate is formed called an alkoxide intermediate. In the second step, hydrogen or protonation is added. The hydrogen ions from an acid source, i.e., HCl, are attacked by the negative charges on oxygens of alkoxide ion intermediate. Therefore, hydrogen atoms are attached to oxygens, and BH_2_ detaches. Thus, the ketonic form of quinone is converted to hydroquinone, finally forming THQ.

##### 2.2.1.2 Esterification of THQ with GA

**Scheme 2:**
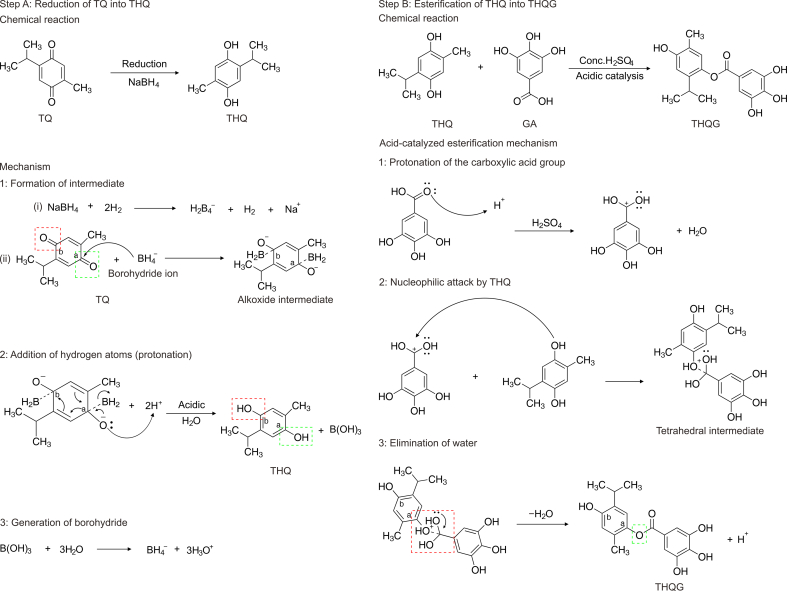
Schematic illustration of the synthesis of thymohydroquinyl gallate-capped gold nanoparticles. Step A: Reduction of thymoquinone (TQ) to thymohydroquinone (THQ) via NaBH_4_. Step B: Esterification of THQ and gallic acid (GA). THQG: thymohydroquinyl gallate.

After the THQ preparation, simple acid-catalyzed esterification was carried out between THQ and GA. 3 g GA, 10 mL THQ, and 0.5 mL conc. H_2_SO_4_ was added to a 50 mL flask dropwise and refluxed for 4 h. After cooling to room temperature, the ester was separated via a separating funnel using distilled water, CCl_4_, and NaHCO_3_ [12]. The product was washed with distilled water and dried on anhydrous magnesium sulfate. The reaction mechanism involves three steps, i.e., protonation of the carboxylic acid group, nucleophilic attack by THQ, and elimination of water (Scheme 2). In the first step, the carboxylic group of GA is protonated, breaking the pi bond in C=O and generating a C^+^–OH entity, also called the carbocation intermediate. A positive charge on the carbon center is called an electrophilic center. Any nucleophilic entity can quickly attack this electrophilic carbon. In the second step, oxygen lone pairs of hydroxyl groups in THQ act as nucleophilic centers towards the electrophilic carbon of carbocation. Hence, a nucleophilic attack by THQ on GA electrophilic carbon generates a tetrahedral intermediate. In the final step, water is eliminated from the intermediate's electrophilic carbon by removing one OH^−^ and one H^+^ of another OH group from the same carbon. A double bond is generated between C and O, forming an ester bond. This final product is called THQG.

##### 2.2.1.3 Synthesis of THQG*–*AuNPs

**Scheme 3:**
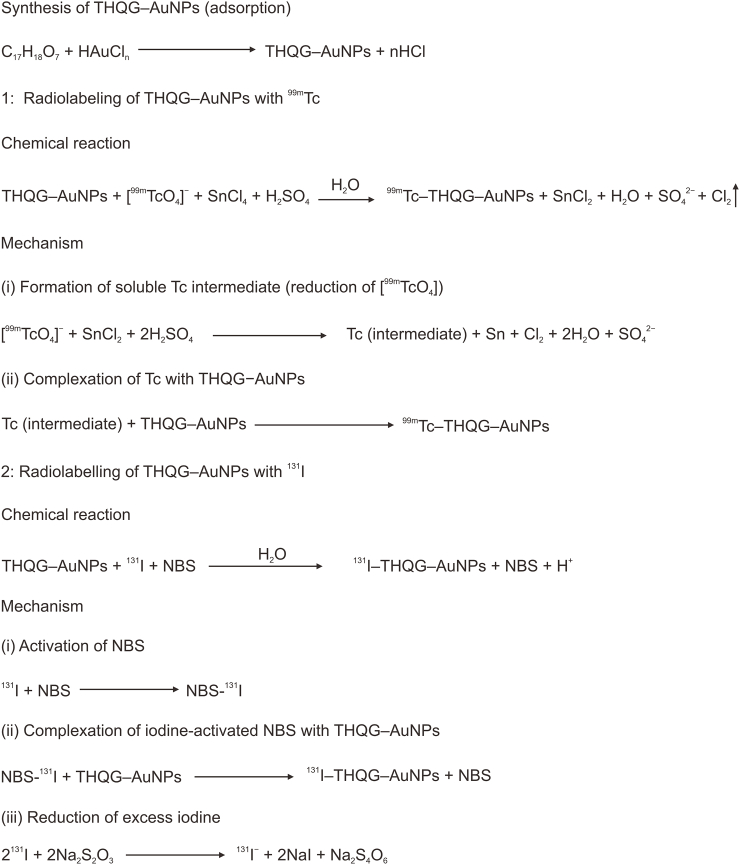
Mechanism of synthesis of thymohydroquinyl gallate (THQG) loaded gold nanoparticles (AuNPs) and radiolabelling with ^99m^Tc and ^131^I. NBS: N˗bromosuccinimide.

THQG–AuNPs were synthesized by reducing HAuCl_4_ with the above-synthesized ester, e.g., THQG. Here, THQG acted as a reducing agent for Au metal and the drug. Therefore, in the loading step, 1.25 mL of 10 mM HAuCl_4_ solution was added to 100 mL DI water and refluxed under magnetic stirring. 0.85 mL ester solution in DI water (1% solution) was then added and refluxed for another 30 min until the vine red color appeared. The Au nanosuspension of the esterified drug was centrifuged at 6000 rpm for 1 h and resuspended again in DI water [18] (Scheme 3).

##### 2.2.1.4 Radiolabeling of THQG–AuNPs with ^99m^Tc

200 μL nanodrug was dissolved in 1 mL distilled water, and 1 mL of 1 M SnCl_2_·2H_2_O was used as a reducing agent. Then, 10 μL H_2_SO_4_ was added to maintain pH 5. Then 10 μL of ^99m^Tc was added to the reaction mixture [19], and the dose for *in vivo* studies was selected according to the body mass index of each animal under investigation. ^99m^Tc was used as a pertechnetate ion (^99m^TcO_4_^−^) as a radiocontrast agent for various organ identification and analysis. SnCl_2_ is oxidized while pertechnetate ion (^99m^TcO_4_^−^) is reduced to label the nanoester drug (THQG−AuNPs) and a soluble ^99m^Tc intermediate. The Tc in the pertechnetate ion is converted to a +4 oxidation state in the presence of SnCl_2_. In the second step, a radiolabelled nanoformulated esterified drug is formed by the THQG−AuNPs complexation with the soluble Tc intermediate obtained from the first step (Scheme 3).

##### 2.2.1.5 Radiolabelling of THQG–AuNPs with ^131^I

100 μL nanodrug was dissolved in 1 mL freshly prepared 1 M phosphate buffer (PBS). 100 μg NBS was added as an oxidizing agent and gently shaken. Then, 10 μL ^131^I solution (5mCi/10 μL) was added. The reaction continued for 1 h, and 1 mL of 1 M Na_2_S_2_O_3_ was added to quench the radioactive ^131^I [20].

**Fig. 1:**
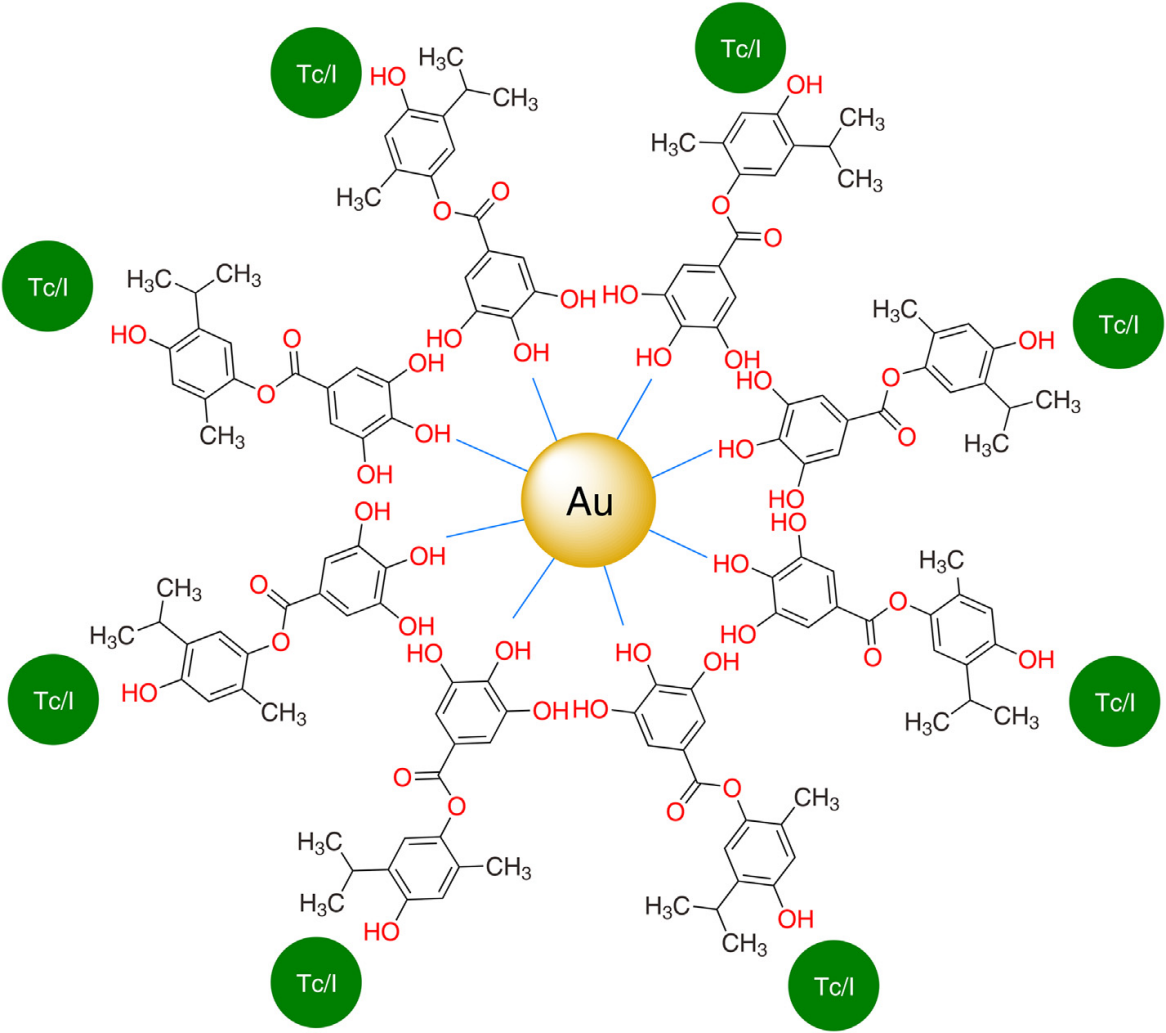
Thymohydroquinyl gallate (THQG)-loaded gold nanoparticles (AuNPs) radiolabeled with either ^99m^Tc or ^131^I. Au: gold; Tc: technetium; I: iodine.

There are three simple steps of ^131^I radiolabelling. In the first step, NBS oxidizes ^131^I, and a ^131^I-activated NBS intermediate is synthesized. In the second step, chelation occurs between the NBS−^131^I intermediate and THQG−AuNPs drug, and a ^131^I−labeled nanodrug complex (^131^I−NBS−THQG−AuNPs) is formed. In the final step, the reaction is quenched to reduce the excess of ^131^I via Na_2_S_2_O_3_. The excess ^131^I is converted to sodium iodide, and the response is quenched from further progression (Scheme 3). The final product is shown in Fig. 1.

##### 2.2.1.6 Chromatographic analysis of ^99m^Tc- and ^131^I-labeled drug

Radiochemical quality control procedures were performed, and the amounts of labeled or hydrolyzed and free drugs were analyzed through chromatography. Instant thin layer chromatography-silica gel (ITLC-SG) and Whatman filter paper number 3 (W.P.No.3) strips of the respective type of chromatography were tested by Gamma counter, i.e., first recorded the counts of the complete long strip and then cut the strip into two as upper half (unbound drug) and the lower half (hydrolyzed drug). The percentage of the free and bound or hydrolyzed drugs was calculated by the following formula [21]:

(1) $$\text{Free}\;\text{drug}=\frac{\text{upper}\;\text{half}\;\text{count}}{\text{total}\;\text{count}}\times 100$$

(2) $$\text{Bound}\;\text{or}\;\text{hydrolyzed}\;\text{drug}=\frac{\text{lower}\;\text{half}\;\text{count}}{\text{total}\;\text{count}}\times 100$$

### 2.3 UV-visible (Vis) spectrophotometric analysis

UV-Vis spectra of TQ, THQG, AuNPs, and THQG−AuNPs were recorded by Spectro quant Pharo 300 MERCK spectrophotometer (MERCK, Darmstadt, Germany). The samples were prepared by dissolving 5 mg of TQ and THQG in 1 mL ethanol and 1 mL THQG−AuNP suspension in deionized water. The samples were first ultrasonicated for 10 min, and then a complete absorption scan of each sample was obtained. Finally, results were plotted graphically using origin software.

### 2.4 Fourier transform infrared spectroscopy (FTIR)

FTIR analysis was done to analyze the functional groups of materials, i.e., TQ, GA, THQG, and THQG−AuNPs. The solvents for non-radiolabeled samples were the same as for UV-Vis analysis; amounts of products were used: ⁓5 μg of each of TQ, GA, and THQG, and 5 μL of THQG−AuNPs suspension. FTIR spectra were recorded on the PerkinElmer FTIR spectrophotometer (PerkinElmer, Waltham, MA, USA) in transmission mode from 4,000 to 500 cm^−1^.

### 2.5 Drug loading efficiency

Loading of THQG on AuNPs was recorded by UV-Vis spectroscopy. THQG−AuNPs were centrifuged at 13,000 rpm for 30 min, and the supernatant was collected and analyzed. The concentration of THQG in the supernatant was quantified at 247 nm (*λ*_max_). The amount of loaded THQG was estimated as the total THQG used minus the THQG quantity in supernatant [22].

(3) $$\text{Supernating}\;\text{efficiency}=\frac{\text{Total}\;\text{amount}\;\text{of}\;\text{THQG}–\text{AuNPs}\;-\;\text{free}\;\text{THQG}–\text{AuNPs}\;\text{in}\;\text{supernatant}\;}{\text{Total}\;\text{amount}\;\text{of}\;\text{THQG}–\text{AuNPs}}\times 100$$

### 2.6 *In vitro* drug release

To estimate the *in vitro* drug release from THQG−AuNPs, the sample and separate (SS) method was used where 10 mL colloidal drug suspension (THQG−AuNPs = 0.085 mg/mL) was added to 100 mL PBS (pH 7.4 and 5.8). The mixture was stirred at room temperature for about 3 h. The drug release was determined via UV˗Vis spectroscopy by taking 3 mL of release media, i.e., PBS at regular intervals, replaced by an equal amount of fresh solvent to the incubation medium.

### 2.7 *In vitro* antineoplastic role of drug

*In vitro,* the antineoplastic role of the drug is observed by cytotoxicity measurements on oral squamous cell carcinoma (OSCC) cell lines (CAL-27). The ability of live cells to convert 3-[4,5-dimethylthiazol-2-yl]-2,5 diphenyltetrazolium bromide (MTT) into formazan is tested using the MTT-colorimetric cytotoxicity assay. CAL-27 cells (5103) are planted in a 96-well culture dish and treated separately for 0, 24, and 48 h with 6.25, 12.5, 25, 50, 100, and 200 μg THQG and THQG−AuNPs. Dimethyl sulfoxide (DMSO) is used as a control. After treatment, each well is filled with 20 μL of MTT solution (5 mg/mL in PBS) and incubated for 2 h. The supernatant is aspirated when the experiment ends. MTT formazan produced by metabolically live cells is solubilized in 100 μL isopropanol. Using a gyratory shaker (KC Engineers Ltd., Haryana, India), the plates are stirred for 30 min, and absorbance is recorded at 595 nm on an analyzer (Infinite M200, Tecan, Männedorf, Switzerland).

### 2.8 *In vivo* biodistribution of radiolabeled drugs in animal specimens

Animal subjects, i.e., female Wister rats, were administered with the radiolabeled drug-loaded AuNPs. Ethical consent was obtained from the Ethical Committee of Bahauddin Zakariya University, Multan, Pakistan (Approval number: 01/926/UREC/2022). In the control group, the rats were normal, while those in the experimental group were cancerous. Both groups were treated with equal volumes, i.e., 1 mL of the Tc-labeled drug and 2 mL of the labeled drug (Tc−THQG, I−THQG, Tc−THQG−AuNPs, I−THQG−AuNPs). The radio-atom activity was corresponding to their body weights. A fresh drug injection was prepared for each rat following its body mass index. After 2 h of tail injection (time required for complete drug biodistribution), each rat was carried to a scanning area, i.e., gamma camera and single-photon emission computed tomography-computed tomography (SPECT-CT; GE Healthcare, Lahore, Pakistan) for static view and the whole-body scan to visualize the distribution of the administered drug. The scheme for type and amount of drug dose with respective radiolabel activity in mCi according to the weight of rats in the control and experimental groups are explained in Table 1. Four rats in each group were named from 1 to 13. Four drug formulations were administered and examined in rats (as presented in Table 1).

**Table 1:** The work plan of *in vivo* biodistribution analysis of radiolabeled drug.

Rat labels	Weight (g)	Drug administered	Drug dose (for injection)	Activity (mCi)
Rat 1 (control)	200	^99m^Tc−THQG	200 μg (THQG)	0.085
Rat 2 (control)	240	^99m^Tc−THQG−AuNPs	200 μL (THQG−AuNPs)	0.103
Rat 3 (control)	210	^131^I−THQG	200 μg (THQG)	2.500
Rat 7 (control)	260	^131^I−THQG−AuNPs	200 μL (THQG−AuNPs)	2.500
Rat 8 (experimental)	225	^99m^Tc−THQG	200 μg (THQG)	0.096
Rat 9 (experimental)	230	^99m^Tc−THQG−AuNPs	200 μL (THQG−AuNPs)	0.099
Rat 10 (experimental)	255	^131^I−THQG	200 μg (THQG)	2.500
Rat 13 (experimental)	270	^131^I−THQG−AuNPs	200 μL (THQG−AuNPs)	2.500

THQG: thymohydroquinyl gallate; THQG–AuNPs: thymohydroquinyl gallate–capped gold nanoparticles.

200 μL drug (THQG−AuNPs) suspension was used to prepare injections for all rats of the same age and approximately the same body weights (200−270 g). The radioactivity dose was separately calculated for each rat according to its body weight, as an overdose or low dose affects the drug visualization. It was calculated by comparing the ^99m^Tc dose used for 7 kg kid as 3 mCi:

^99m^Tc dose used for 7 kg (7,000 g) kid = 3 mCi

(4) $${{}_{}}^{99m}\mathrm{Tc}\;\text{dose}\;\text{used}\;\text{for}\;X\;\text{(g)}\;\text{rat}=\frac{3X}{7000}=Y\;(\text{mCi})$$

Where *X* stands for the relevant digital value of rat weight, while Y stands for dose in mCi for rat. The above-calculated dose was adjusted by a milli Curie counter before activating the drug formulation. ^131^I-dose was not calculated according to the rat's body weight as it has a longer half-life (8 days) and is less toxic than ^99m^Tc (i.e., a shorter half-life of 6 h). Hence, 10 μL (1 drop = 2.5 mCi, dose measured via milli Curie counter) was generally added to injecting drug radiolabeled with ^131^I.

A Symbia Evo Excel gamma camera (Siemens Healthineers, Erlangen, Germany) was used at leap mode (low energy mode), and 300K counts were recorded for each rat under 3.25× magnification for a static view of drug distribution in the rat body. The CT-scan apparatus (GE Healthcare, Lahore, Pakistan) was used for the whole-body scan via SPECT-CT to visualize the maximum number of drug-deposited organs in the rat body. After the whole body scan, rats were dissected, and their organs were taken out and preserved in formalin for further activity measurement by gamma counter for a 30 s cycle per tray mode. The activity counts were evaluated as the percentage of drug accumulation in the respective organ. This gamma counting was done for all rats after their whole-body scans. The counts of all organs were summed up to a total value, and the drug/activity percentage was calculated as follows:

Totalcounts=(organ1counts+organ2counts+….)

(5) $$\;\text{drug}\;\text{deposition}\;\text{in}\;\text{each}\;\text{organ }(\%)=\frac{\text{counts}\;\text{in}\;\text{each}\;\text{organ}}{\text{total}\;\text{counts}\;\text{of}\;\text{all}\;\text{organ}}\times 100$$

The three approaches, i.e., spotted view in gamma camera, whole body scan, and drug deposition (%) in each organ, confirmed the drug biodistribution in the rat's body. The drug can thus be reported for biodistribution in control and experimental rat groups and has an antineoplastic role in the organ with maximum deposition.

## 3 Results and discussion

**Fig. 2:**
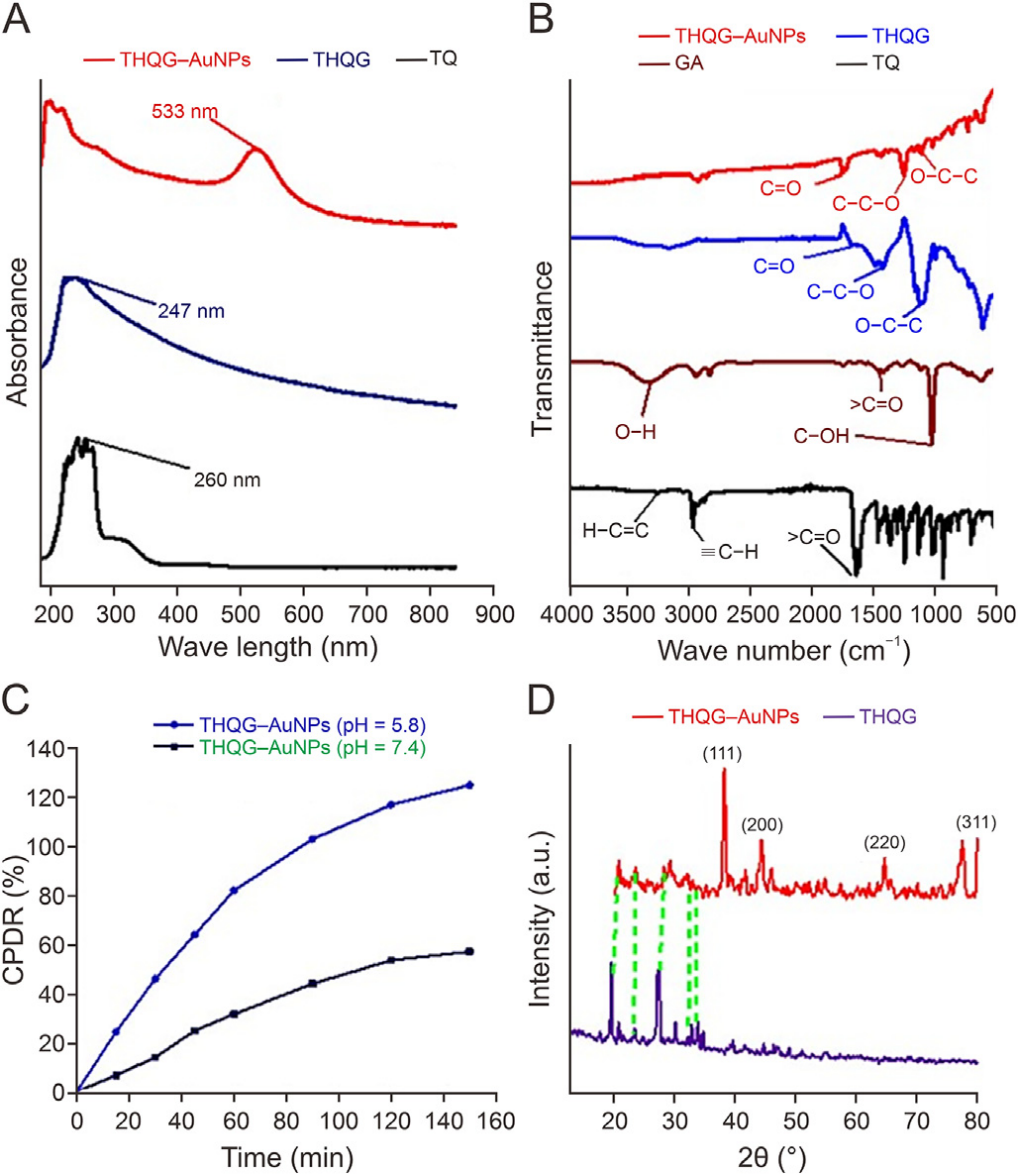
Structural characterization of synthesized materials. (A) UV-Vis spectra of thymoquinone (TQ), thymohydroquinyl gallate (THQG), and thymohydroquinyl gallate–capped gold nanoparticles (THQG–AuNPs). (B) Fourier transform infrared spectroscopy (FTIR) spectra of TQ, GA, THQG, and THQG−AuNPs. (C) *In vitro* drug release at different pH (physiological ⁓7.4 and tumor microenvironment ⁓5.8). (D) X-ray diffraction of ester and ester–loaded AuNPs. CPDR: cumulative percentage of drug release.

The UV-Vis spectrum of TQ and THQG authenticates the binding of THQ and GA (Fig. 2A). The *λ*_max_ of TQ is at 260 nm while the esterified product shifts to 247 nm. THQG−AuNPs are synthesized by reducing HAuCl_4_ salt solution with polyphenols. The maximum absorbance of the THQG−AuNPs nanodrug is ≈ 533 nm. The modulation in AuNP photophysical attributes corresponds to the absorption band of noble metals' surface plasmon resonance property [23]. The bandwidth and *λ*_max_ depend on several factors, such as dielectric surroundings, agglomeration among particles, nanoparticle morphology, and formation of secondary metabolites during the synthesis [24,25].

FTIR spectrum (Fig. 2B) of TQ exhibits carbonyl stretching at 1655 cm^−1^, a characteristic band of 1,4–benzoquinone. C–H stretch of aliphatic moieties is observed at 2966 cm^−1^. C–H stretching of the vinyl group appears at 3038 cm^−1^. The band at 1640 cm^−^^1^ corresponds to >C=O stretching. C=C band is less intense than both carbonyl bands and C=C stretching of 1,4−cyclohexadiene, with no isopropyl or methyl substituents [26].

GA shows O–H stretching at 3325 cm^−^^1^, previously reported at 3496 cm^−^^1^ and 3282 cm^−^^1^. A signal at 1747 cm^−^^1^ is accompanied by a C=O stretch of the carboxylic group on the aromatic ring, and bands at 1449 cm^−^^1^ and 1556 cm^−^^1^ are associated with the aromatic ring substitutions [27]. In the fingerprint region, an intense band at 1255 cm^−^^1^ corresponds to a C–O stretch (1210–1320 cm^−^^1^) [28]. In esters, three stretching vibrations of C=O, O–C–C, and C–C–O observed at 1715–1730 cm^−^^1^, 1250−1310 cm^−^^1^, and 1100−1130 cm^−^^1^, respectively [29]. In the present study, THQG shows IR bands at 1248 cm^−^^1^ and 1122 cm^−1^, corresponding to C–C–O stretch of ester. These results confirm the ester synthesis.

THQG and THQG–AuNPs both show C=O stretch at 1746 cm^−^^1^ and 1748 cm^−^^1^ respectively. At 1465 cm^−^^1^, the aromatic stretch depicts a slightly intense band of THQG−AuNPs. C–C–O stretch is observed at 1381 cm^−^^1^ for THQG−AuNPs, showing a shift from 1248 cm^−^^1^ of THQG without conjugation to Au nanoparticles. According to the rule of three, the O–C–C stretch is marked at 1115 cm^−^^1,^ also showing a shift from 1248 cm^−^^1^ of THQG before AuNPs attachment [29]. O–H bending appears at 3342 cm^−^^1^, confirming the successful formation of the complex.

According to the above equation, the calculated drug loading is 96.8%. Slow drug release at pH 7.4 while fast release at target site enhance the drug efficacy (Fig. 2C). The cumulative percentage of drug release (CPDR%) is calculated at physiological pH 7.4 and tumor microenvironment pH 5.8 for 150 min. After 90 min, CPDR% at pH 7.4 is 44%, while at pH 5.8, it is 103%, suggesting that all drug is released at tumor-related pH.

The crystal structure of THQG and THQG−AuNPs is determined from X-ray diffraction (XRD) (Bruker D8 Advance, Billerica, MA, USA) (Fig. 2D). The Scherrer equation correlates micrometer-sized particulates, or crystallites, in a solid substance to the peak broadening in the diffraction pattern (Equation (6)). The Scherrer equation uses the width of the broadest XRD signal for a specific sample to estimate the crystal grain size. The equation is given as:

(6) $$D=\frac{k\lambda }{\beta \;\cos\;\theta }$$

where *D* is the particle size in nm, k represents Scherrer constant (0.9), *λ* is the X-ray wavelength of Cu Kα1 radiation (1.5406 nm), *β* is FWHM (radians), and *θ* is peak position (radians). The average particle size of THQG and THQG–AuNPs is calculated from peak width obtained on the diffractometer, i.e., about 147.63 nm and 5.634 nm, respectively.

**Fig. 3:**
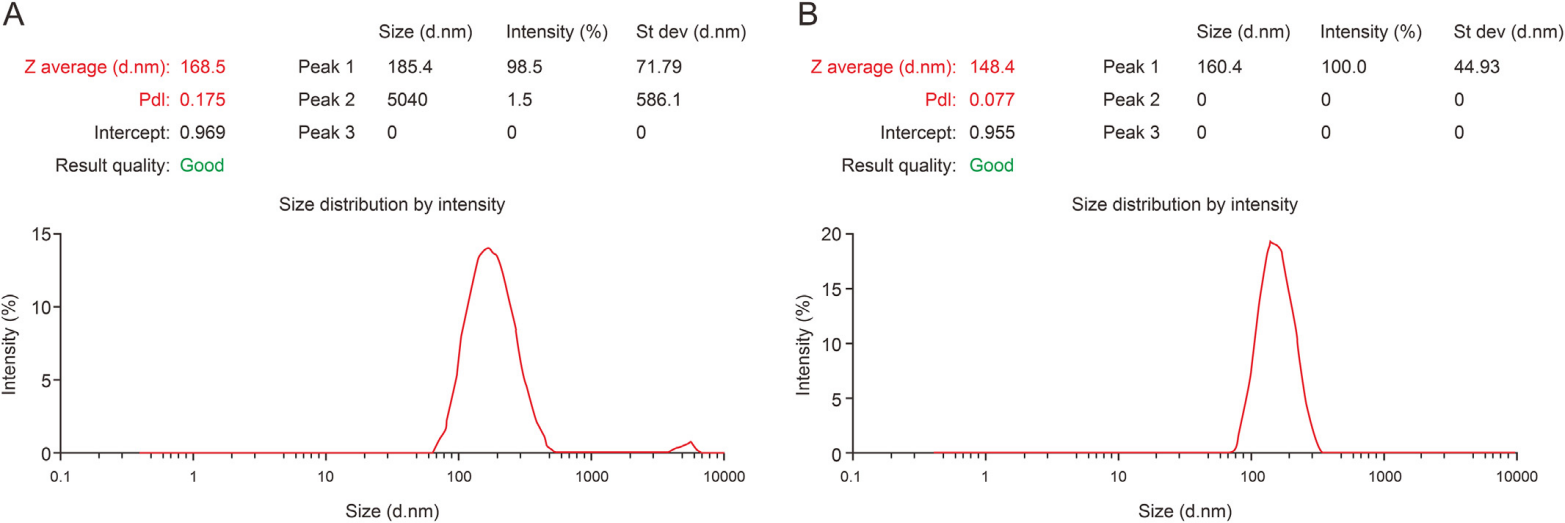
Graphical representation of Zeta potential of (A) thymohydroquinyl gallate (THQG) and (B) thymohydroquinyl gallate*–*capped gold nanoparticles (THQG–AuNPs).

In the Zeta potential graph (Fig. 3A), the average particle size for THQG is 168.5 nm, while that of THQG−AuNPs is 148.4 nm (Fig. 3B).

**Fig. 4:**
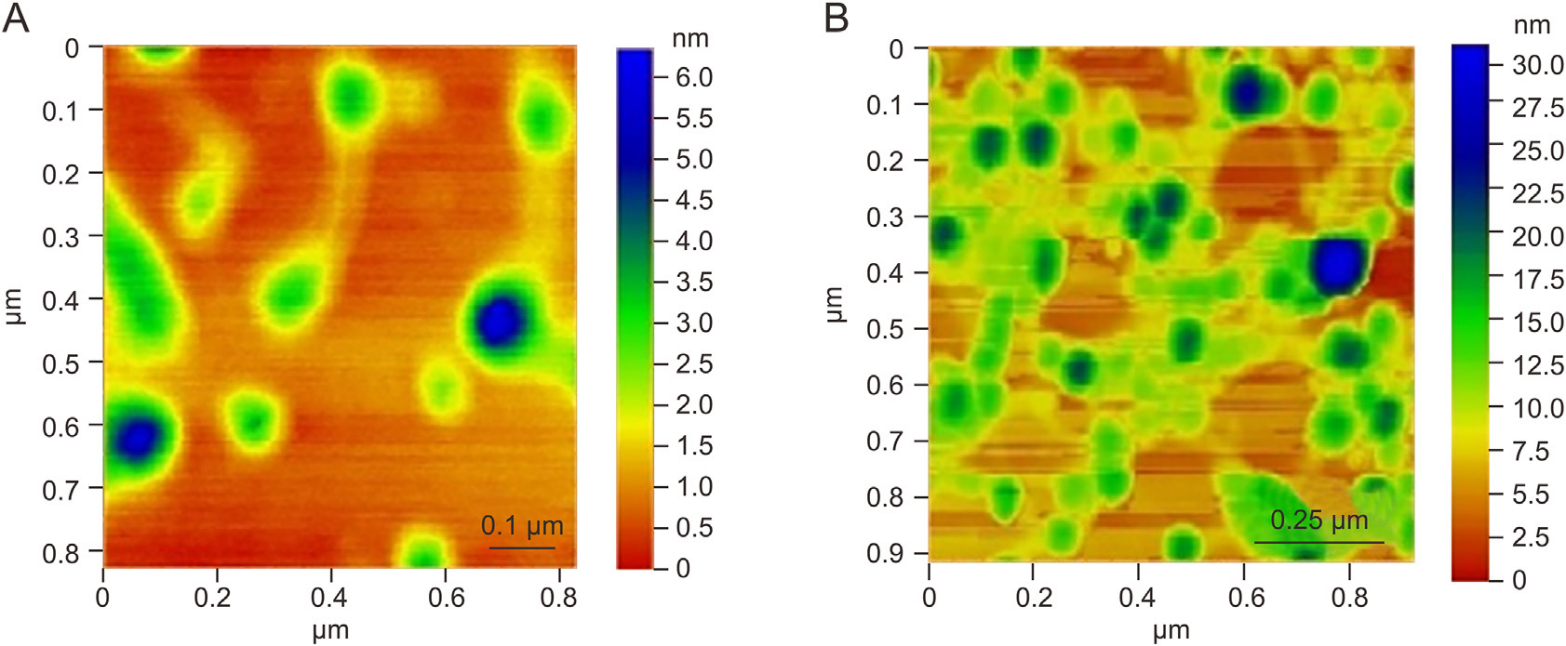
Atomic force microscopy (AFM) of thymohydroquinyl gallate*–*capped gold nanoparticles (THQG–AuNPs). (A) at 0.1 μm, and (B) at 0.25 μm.

**Fig. 5:**
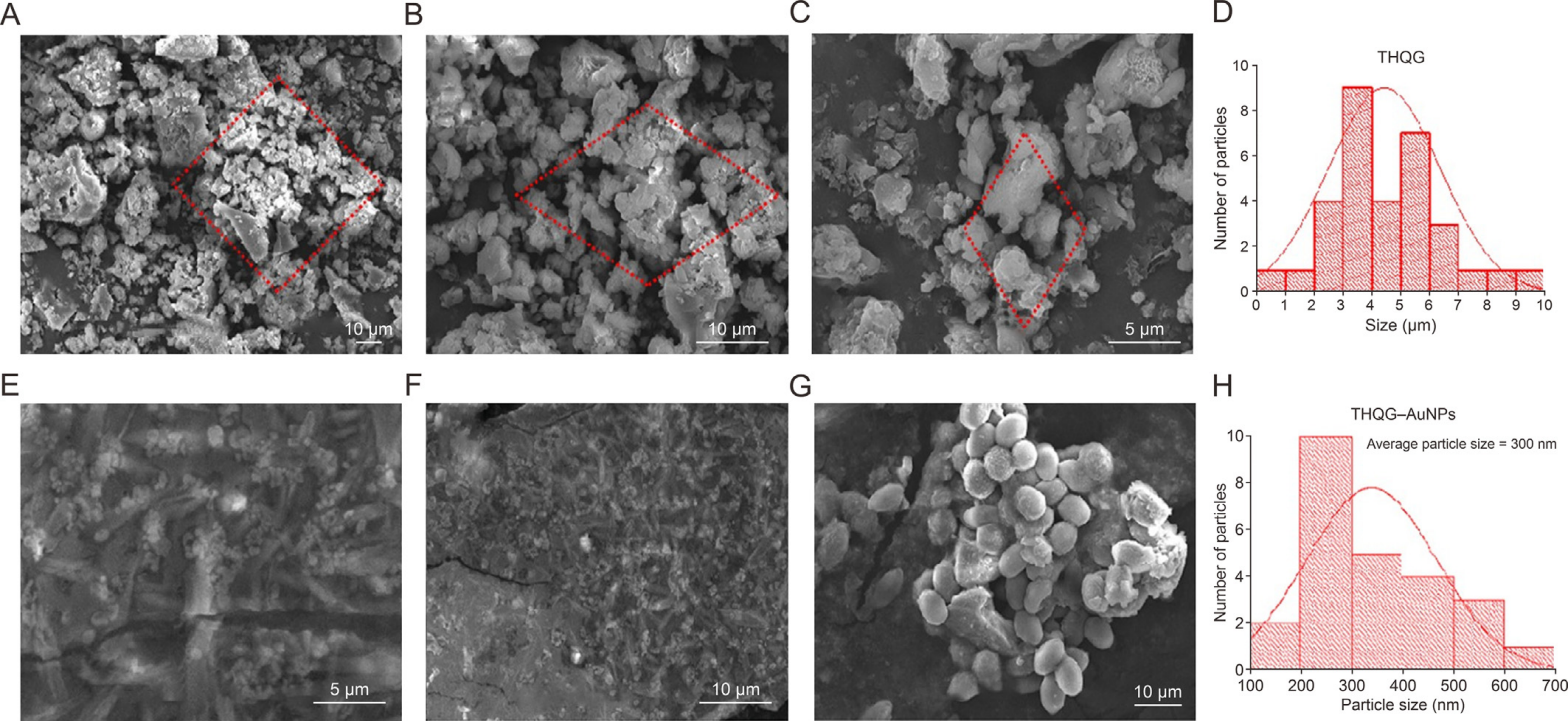
(A*–*D) Scanning electron microscopy (SEM) visual and graphical representation of thymohydroquinyl gallate (THQG). (E*–*H) SEM visual and graphical representation of thymohydroquinyl gallate*–*capped gold nanoparticles (THQG–AuNPs).

Atomic force microscopy (AFM; Bruker) is used to evaluate the dispersion of particles and their surface roughness (Fig. 4). The data under AFM explorer via the non-contact and contact switching is evaluated by the standard probes for two switching modes. Image resolution is kept between 500 and 1,000 pixels, and scan speed is 1–5 μm/s. The sample is prepared using the synthetic protocol for citrate-capped and epigallocatechin gallate-capped AuNPs. Scanning electron microscopy (Jeol, Tokyo, Japan) images of THQG ester are obtained after placing the thin layer on the glass plate and analyzed using ImageJ and origin software. A standard distribution curve generates a histogram and determines the average particle size (Figs. 5A–D). The particle size of THQG is calculated as 4.5 μm which is due to particles' aggregation. At the same time, that of THQG−AuNPs is 253.6 nm from statistical histogram data analysis and is depicted in Figs. 5E–H. These results are consistent with AFM images.

In pharmacology, a compound's potency is measured by the half-maximal effective concentration (EC_50_), which is the drug's concentration that produces a reaction halfway between the baseline and the maximum. A compound's potency is inversely proportionate to its EC_50_ value [30].

**Fig. 6:**
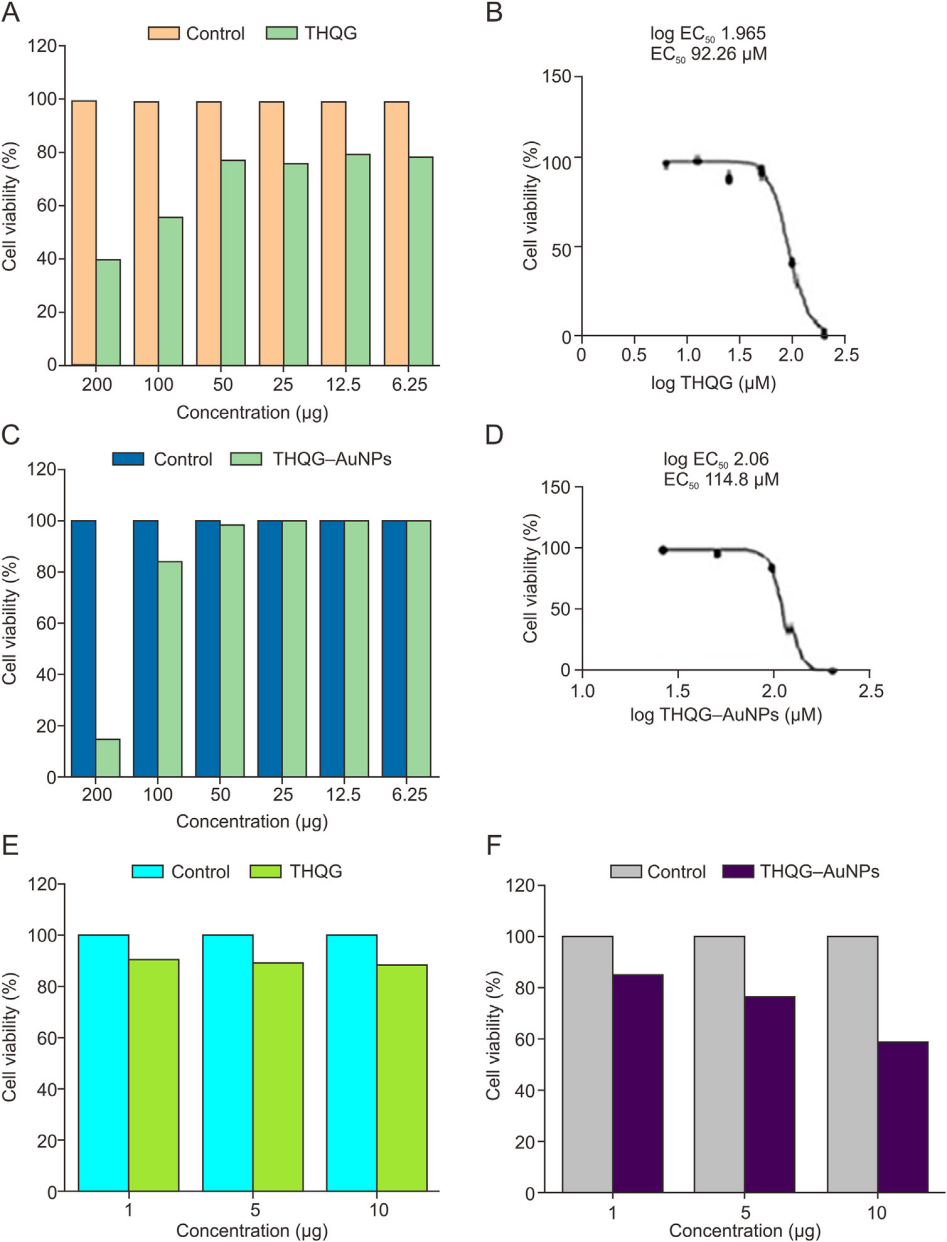
Cell viability results via 3-[4,5-dimethylthiazol-2-yl]-2,5 diphenyl tetrazolium bromide (MTT) assay. (A) Cell viability test of thymohydroquinyl gallate (THQG) at different concentrations. (B) The relationship between log of drug concentration and cell viability. (C) Cell viability test of thymohydroquinyl gallate*–*capped gold nanoparticles (THQG–AuNPs) at different concentrations. (D) The relationship between log of drug concentration and cell viability on CAL 27 cells. (E, F) Cytotoxicity of (E) THQG ester and (F) THQG–AuNPs on MCF 7 breast cancer cell lines.

A graphical representation of cytotoxicity acquisition via MTT assay of THQG and that of THQG−AuNPs on CAL 27 cells is given in Figs. 6A and C. When cells are treated with the increasing concentration of THQG, various concentration-dependent results are observed. Compared with control, the cell viability of THQG is up to 80% from 6.25 μg to 50 μg. Increasing concentration above 50 μg shows a gradual decrease in cell viability. At 100 μg, it reduces to 57%, and further increasing to 200 μg decreases the cell viability to 40%. Fig. 6B shows the relationship obtained between the micromolar concentration of the drug versus cell viability. From log 0.7 μM to log 1.7 μM, cell viability is 100%. It gradually decreases with a further increase in concentration. At log 2 μM, the viability reduces to 42%, and at 2.4, it becomes 0%. EC_50_ is recorded as 92.26 μM while log EC_50_ is 1.965 μM. These findings verify the expected theranostic roles of prodrug composite, i.e., EPR, doubled antineoplastic role of drug conjugate.

For THQG–loaded AuNPs, the cytotoxicity test is performed, and the cell viability percentage is recorded (Fig. 6C). The cell viability graph shows that for 6.25, 12.5, 25, and 50 μg, the cell viability is up to 100%. At 100 μg, it reduces to 80%; at 200 μg, it decreases to 15%. Fig. 6D represents the cell viability versus log drug concentration. At log 1.3 μM to log 2 μM, cell viability remains 100%, i.e., no drug effect at these concentrations. A further increase to 2.06 μM reduces cell viability to 80%, and the cell viability reduces to 0% at log 2.3 μM meaning no viable cells are present at this drug concentration. The log EC_50_ is obtained as 2.06 μM and EC_50_ as 114.8 μM.

The cytotoxicity of THQG ester drug is tested on MCF 7 breast cancer cell lines (Fig. 6E). The representation shows that at 1, 5, and 10 μg of THQG, the cell viability reduces to 90%. When the same drug is loaded onto AuNPs, it penetrates the cell through the cell membrane and shows more antineoplastic activity than simple THQG. Fig. 6F reveals that at 1 μg, cell viability is 80%. At 5 μg, cell viability is reduced to 70%. Further increase in drug concentration to 10 μg decreases the cell viability to 55%.

**Fig. 7:**
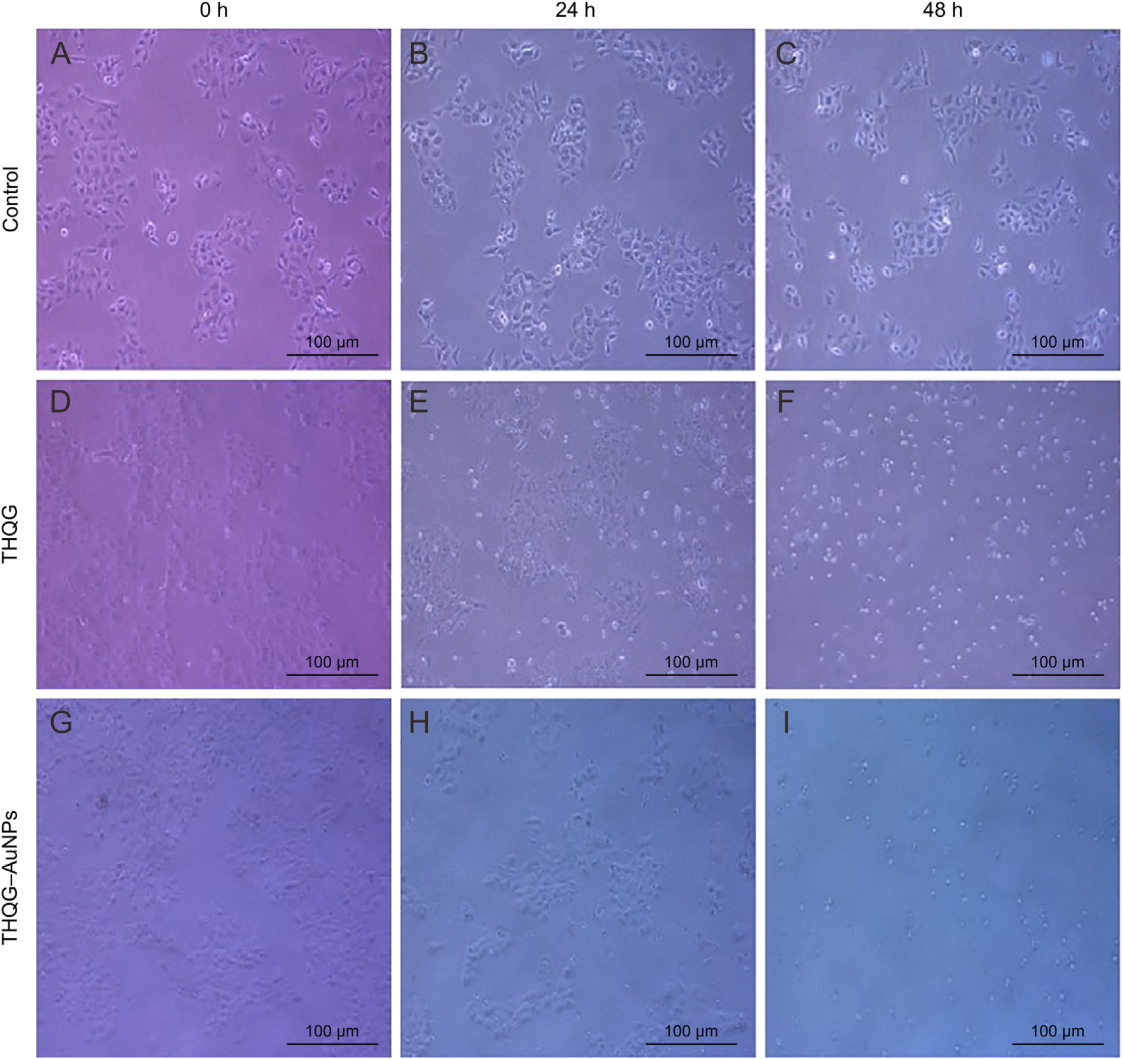
Microscopic visualization of antineoplastic activity of the drug at 0 h, 24 h and 48 h post drug treatment. (A*−*C) Control group, (D*−*F) cells treated with thymohydroquinyl gallate (THQG), and (G*−*I) cells treated with thymohydroquinyl gallate–capped gold nanoparticles (THQG–AuNPs).

This microscopic visualization of CAL 27 cell lines corresponds to the above mentioned cell viability graphs. Figs. 7A−C represents cell presence in the control group, where cell viability remains 100% as no anticancer drug is added to the control. Hence, Fig. 7A shows control cells at day 0, Fig. 7B depicts control cells 24 h later, and Fig. 7C shows the cells in the control group at 48 h. As no drug was added in control group, there was no reduction observed in shape or number of viable cells in control group after 48 h. Figs. 7D–F depicts the group of cells treated with THQG. Fig. 7D represents viable cells at day 0 before drug treatment. After 24 h, cells start dying, the number of viable cells decreases (Fig. 7E). Fig. 7F illustrates cells after 48 h of drug treatment. The number of viable cells significantly decreases, with pronounced morphological changes. The cells treated with THQG−loaded AuNPs are shown in Figs. 7G−I. Fig. 7G shows cells at day 0 before drug treatment, where cells are alive. After 48 h (Fig. 7I, fewer cells are utterly absent. Also it is obvious from the results that ester drug when administered with AuNPs exhibited more effectively and resulted in more cells death after 48 h.

Radiolabeled drugs are chromatographically inspected by recording the activity counts on the upper and lower half, showing the percentage labeling of initial and unbound drugs. Both activities, i.e., ^99m^Tc and ^131^I, are up to 90% calculated by Equations (1) and (2), and the conditions are given in Table 2, Table 3.

**Table 2:** Chromatographic inspection of ^99m^Tc labeled samples.

Sr. No.	Sample	Kits	Stationary phase	Mobile phase	Free pertechnetate (%)	HR ^99m^Tc (%)
1	^99m^Tc–TQ	MDP	W.P.No.3	0.9% NaCl	7.40	92.27
2	^99m^Tc–TQ	MDP	W.P.No.3	Acetone	5.79	93.64
3	^99m^Tc–TQ	DMSA	TLC˗SG	Acetone	9.11	90.96
4	^99m^Tc–TQ	MIBI	ITLC˗SG	Ethanol	4.20	96.07
5	^99m^Tc˗THQG	MDP	W.P.No.3	0.9% NaCl	3.08	96.80
6	^99m^Tc˗THQG	MDP	W.P.No.3	Acetone	5.15	94.01
7	^99m^Tc˗THQG	DMSA	TLC˗SG	Acetone	2.27	97.99
8	^99m^Tc˗THQG	MIBI	ITLC˗SG	Ethanol	5.46	95.00
9	^99m^Tc˗THQG˗Au	MDP	W.P.No.3	0.9% NaCl	3.94	95.06
10	^99m^Tc˗THQG˗Au	MDP	W.P.No.3	Acetone	4.00	96.00
11	^99m^Tc˗THQG˗Au	DMSA	TLC˗SG	Acetone	2.24	98.10
12	^99m^Tc˗THQG˗Au	MIBI	ITLC˗SG	Ethanol	2.00	97.54

TQ: thymoquinone; THQG: thymohydroquinyl gallate; THQG–AuNPs: thymohydroquinyl gallate–capped gold nanoparticles; MDP: methylene diphosphonate; DMSA: dimercaptosuccinic acid; MIBI: methoxyIsobutylIsomitrile; W.P.No.3: Whatman filter paper number 3; TLC-SG: thin layer chromatography-silica gel; ITLC-SG Instant thin layer chromatography-silica gel.

**Table 3:** Chromatographic inspection of ^131^I labeled samples.

Sr. No.	Sample	Kits	Stationary phase	Mobile phase	Free ^131^I activity (%)	Bound ^131^I activity (%)
1	^131^I–TQ	MDP	W.P.No.3	0.9% NaCl	13.97	86.06
2	^131^I–TQ	MDP	W.P.No.3	Acetone	13.60	86.15
3	^131^I–TQ	DMSA	TLC˗SG	Acetone	12.59	87.00
4	^131^I–TQ	MIBI	ITLC˗SG	Ethanol	14.03	85.07
5	^131^I–THQG	MDP	W.P.No.3	0.9% NaCl	13.81	85.90
6	^131^I–THQG	MDP	W.P.No.3	Acetone	13.39	86.90
7	^131^I–THQG	DMSA	TLC˗SG	Acetone	14.60	87.70
8	^131^I–THQG	MIBI	ITLC˗SG	Ethanol	15.33	84.98
9	^131^I–THQG–AuNPs	MDP	W.P.No.3	0.9% NaCl	9.69	90.13
10	^131^I–THQG–AuNPs	MDP	W.P.No.3	Acetone	7.97	91.80
11	^131^I–THQG–AuNPs	DMSA	TLC˗SG	Acetone	7.17	92.67
12	^131^I–THQG–AuNPs	MIBI	ITLC˗SG	Ethanol	8.17	91.38

TQ: thymoquinone; THQG: thymohydroquinyl gallate; THQG–AuNPs: thymohydroquinyl gallate–capped gold nanoparticles; MDP: methylene diphosphonate; DMSA: dimercaptosuccinic acid; MIBI: methoxyIsobutylIsomitrile; W.P.No.3: Whatman filter paper number 3; TLC-SG: thin layer chromatography-silica gel; ITLC-SG Instant thin layer chromatography-silica gel.

An *in vivo* study was carried out on albino rats. For each type of labeled drug, a drug injection is prepared by the procedure mentioned earlier. The drug biodistribution is observed in the tail under a gamma camera after 2–3 h post-administration. Drug dose (μg or μL), labeling dose (mCi), and injection volume are selected as per the body mass index of each rat. The spotted view of rats is recorded under a gamma camera, and the whole body scan is done under a TOMO-CT/CT scan (GE Healthcare, Lahore, Pakistan).

**Fig. 8:**
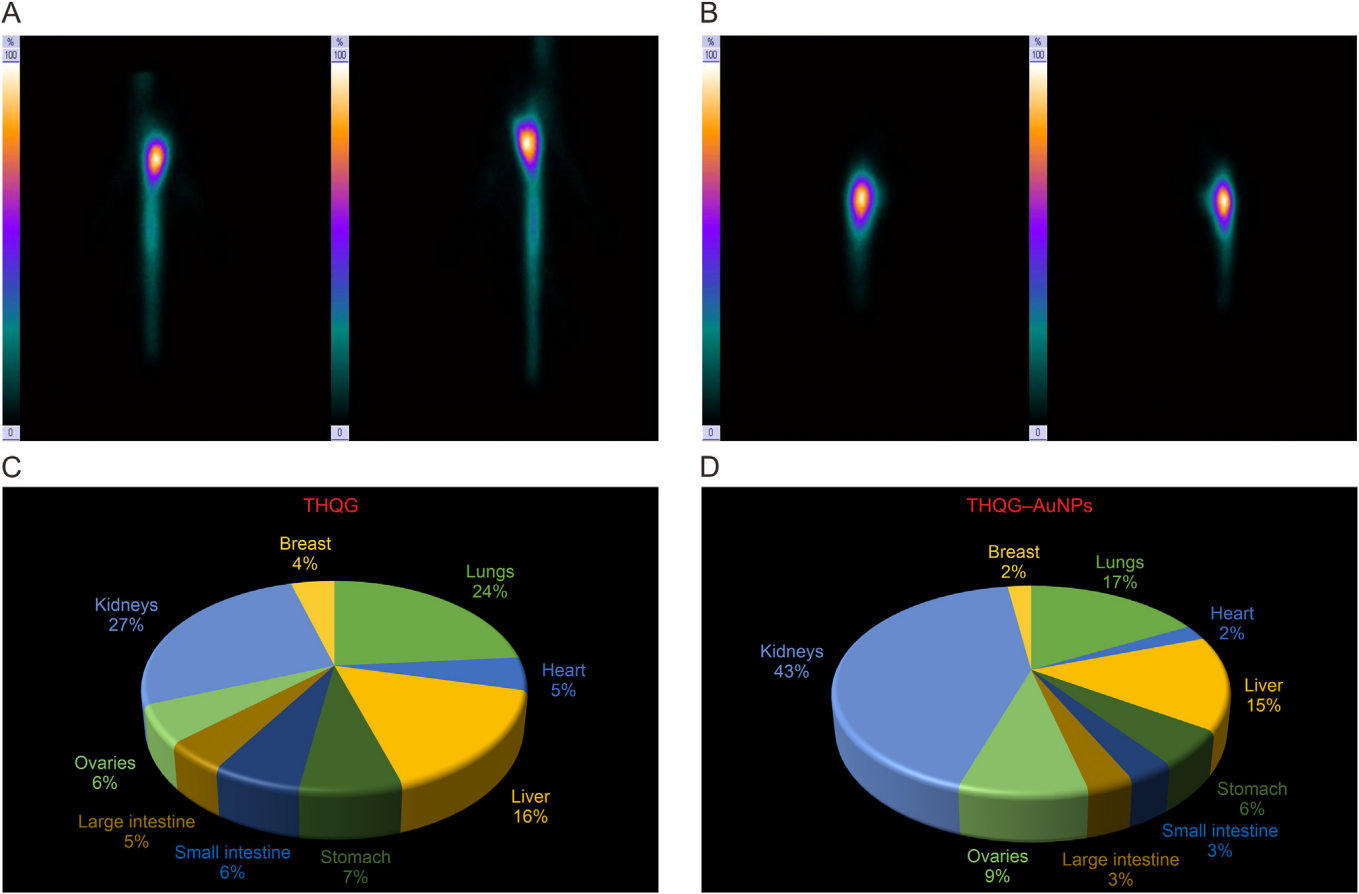
Gamma camera scan. (A) spotted view of Rat 1, (B) spotted view of Rat 2, (C) biodistribution in Rat 1, and (D) biodistribution in Rat 2. THQG: thymohydroquinyl gallate; THQG–AuNPs: thymohydroquinyl gallate–capped gold nanoparticles.

Figs. 8A and B shows the spotted views of rat 1 and rat 2 (control rats). In Fig. 8A, the anterior and posterior view of rat 1 (^99m^Tc−THQG) suggests the drug deposition maximally in the lungs, and the location of the bright spot refers to maximum activity. In Fig. 8C, the biodistribution chart shows the highest kidney and lung activity. Fig. 8B represents maximum activity as the brightest spot in the whole abdomen, referring to the kidneys. Fig. 8D shows a biodistribution chart of rat 2 (^99m^Tc−THQG−AuNPs). The maximum activity is seen in the kidneys and lungs. The gold-loaded drug is radiolabeled and administered. Drug retention is more than that for un-loaded medicines. NP formulation penetrates and stays maximum in the kidneys.

**Fig. 9:**
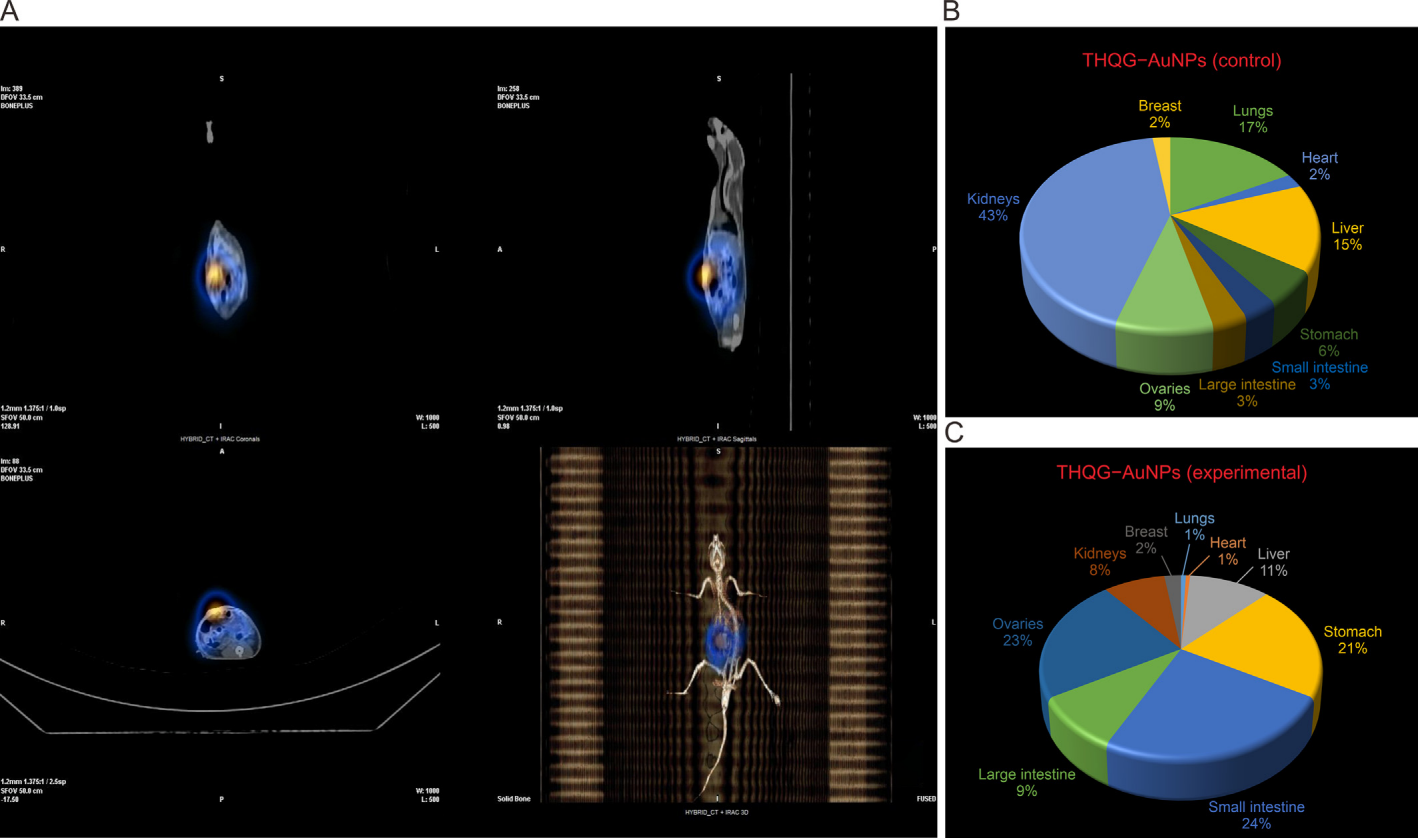
*In vivo* distribution of drugs in different organs of rats. (A) Single-photon emission computed tomography-computed tomography (SPECT-CT) of Rat 9. (B, C) Comparison of *in vivo* biodistribution of Rat 2 (control) (B) and Rat 9 (experimental) (C), respectively. THQG–AuNPs: thymohydroquinyl gallate–capped gold nanoparticles.

Fig. 9A shows the static view and whole body scan of experimental rat 9, whose control is rat 2. Figs. 9B and C compare the biodistribution of ^99m^Tc−THQG−AuNPs in control and experimental rats. The biodistribution chart shows that the same drug is distributed differently in different metabolic bodies, i.e., normal and cancerous. The control rat shows maximum drug deposition in the kidneys and lungs. In contrast, the experimental rat exhibits full drug deposition in other sites, i.e., the highest in the small intestine, ovaries, and stomach.

**Fig. 10:**
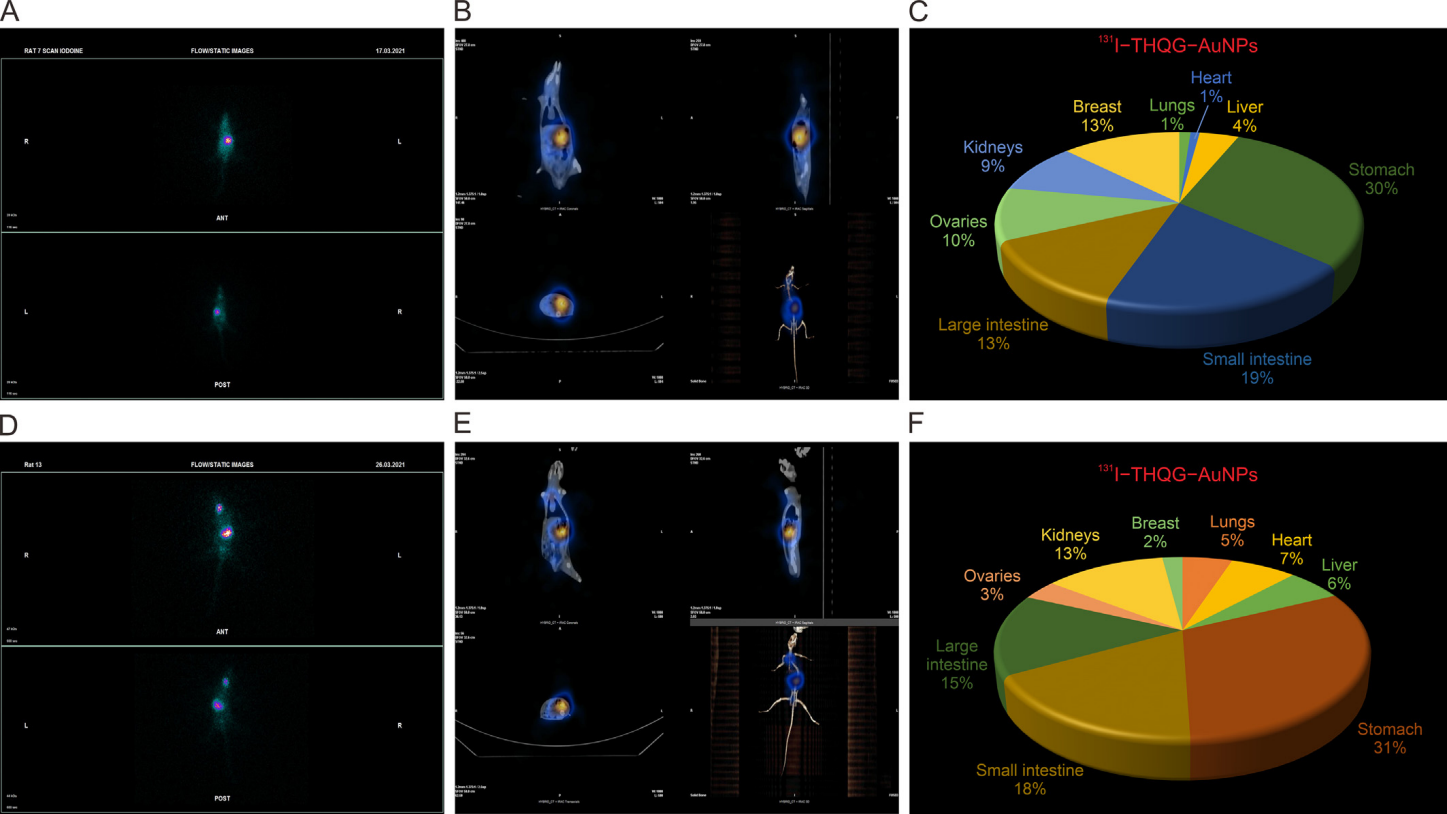
The static view of the radioactive spot representing the actual location of the organ of maximum drug deposition and the whole body scan of Rat 7 (control). (A) Static anterior and posterior views captured in gamma camera. (B) Single-photon emission computed tomography-computed tomography (SPECT-CT) (whole*–*body scan). (C) *In vivo* biodistribution of the drug in rat body. (D) Static anterior and posterior views of Rat 13 (experimental). (E) SPECT*–*CT (whole*–*body scan). (F) *In vivo* biodistribution of the drug in rat body.

Fig. 10A shows the static view of the radioactive spot representing the actual location of the organ of maximum drug deposition, i.e., the stomach in rat 7 (control). Fig. 10B indicates the whole body scan of rat 7 given ^131^I−THQG−AuNPs. This is an average rat with no cancer induced. This rat is dissected, and the organs-specific analysis is carried out via gamma counter (Fig. 10C). Biodistribution percentage data is plotted as a pie chart showing maximum drug or radioactivity deposition in the stomach and small intestine. Rat 13 is the experimental rat and is given ^131^I−THQG−AuNPs. Fig. 10D is the static view of the rat body showing maximum drug deposition in the stomach followed by the small intestine and then the large intestine. Fig. 10E describes a whole body scan of rat 13, where significant activity appears in the stomach. After dissection, the organ biodistribution data is obtained under the gamma counter (Fig. 10F), representing as a pi-chart plot showing maximum deposition of drug in stomach.

Research limitations encountered include that cancer induction involves an extensive period and complete care for the animals under study, then deciding the sufficient dosage of the cancer, inducing drug and antineoplastic drug for each animal corresponding to its body weight, complete animal handling under gamma camera and SPECT, and finally the proper dissection, preservation of organs, blood and urine, and measurements of radioactivity counts in a gamma counter for each organ. Despite many challenges in the proposed study, there are many prospects directed for the theranostic investigation of new and better combinations of two or even more antineoplastic drugs together, acting at their specific targets separately and presenting a combined effect as innovative and the most potent theranostic compounds for the first time. However, several side effects, such as hair and nail loss, are usually seen in conventional chemotherapeutic approaches. These side effects may be less due to the herbal ingredients used in our research plan. Also, the shortened treatment period by novel drugs derived from natural resources may help patients recover earlier than conventional methods. Also, the proposed diagnostic approach can be helpful in monitoring and tracing the drug deposition in the targeted organ visually and help manage drug dosage, recovery, and therapeutic time for patients.

## 4 Conclusions

Two forms of potent antineoplastic drugs, i.e., ^99m^Tc−THQG−AuNPs and ^131^I−THQG−AuNPs, are synthesized. Using more than one drug with the same physical therapeutic effect can be more beneficial in reducing drug dosages and shortening the period of chemotherapy procedure, unlike conventional methods, which are much more tricky for cancer patients. A trial to develop a novel antineoplastic agent sourced from natural resources is conducted. Two drugs possessing antineoplastic roles are linked together chemically via an ester bond to create a conjugate that gets separated inside the tumor microenvironment via esterase enzyme and can perform enhanced antineoplastic roles, i.e., two anticancer drugs working together separately. AuNPs as drug nanocarriers are developed for drug delivery to target sites via the prodrug itself being used as a reducing agent towards Au metal atoms during AuNPs synthesis (plus drug loading), and no linker is needed to load the drug or to synthesize AuNPs. Polyphenolic ester-capped AuNPs are synthesized as esterified prodrug-loaded AuNPs for TDD in animal models and further followed by ^99m^Tc and ^131^I radiolabelling for drug biodistribution and organ-specific bioimaging of drug deposition in animal models visualized via gamma camera, TOMO, and SPECT. Also, the radionuclide labels used here to label the whole prodrug ester loaded onto AuNPs for RIT, RIT-induced EPR effect, and no need of activity dose administration as conventionally given to patients orally or intravenously for organ visualization were found highly worth using as they produced multiplex benefits including above technique. The targeted delivery potentiated the selective cytotoxicity of THQG for cancerous tissues. This work reveals the enhanced and doubled antineoplastic role of prodrug composite post-administration in cell lines and organ-specific biodistribution in animal models. Our prospects include more detailed and every-minute studies for carefully analyzing further benefits of prodrug composite technology, i.e., crossing the blood−brain barrier, thorough treatment of tumors in animal models, reducing time, doses, and frequency of doses required for a complete prodrug treatment course of chemo theranostics.

## CRediT authorship contribution statement

**Munaza Batool:** Methodology, Writing – original draft. **Batool Fatima:** Supervision, Conceptualization. **Dilshad Hussain:** Data curation, Validation. **Rubaida Mahmood:** Resources, Formal analysis. **Saeed Akhter:** Resources, Investigation. **Muhammad Saqib Khan:** Resources, Validation. **Saadat Majeed:** Visulization, Validation. **Muhammad Najam-ul-Haq:** Conceptualization, Writing – review & editing.

## Declaration of competing interest

The authors declare that there are no conflicts of interest.
